# Supplementary material for: Evolution of cross-tolerance in Drosophila melanogaster as a result of increased resistance to cold stress
Source: Sci Rep. 2022 Nov 14;12:19536. doi: 10.1038/s41598-022-23674-z (PMC9663562; doi:10.1038/s41598-022-23674-z)
Supplement: Supplementary file 1 — Supplementary Information. [file 41598_2022_23674_MOESM1_ESM.docx]

**Evolution of cross-tolerance in *Drosophila melanogaster* as a result of increased resistance to cold stress**

Karan Singh^a*^, Manas Arun Samant^b^, Nagaraj Guru Prasad^c^

^a*^Current Affiliation:

NYU Grossman School of Medicine, Department of Cell Biology, 595 Medical Science Building

550 First Ave, New York, NY 10016, USA

E-mail: Karan.Singh2@nyulangone.org

Ph: +1- 212-263-5327

^a*^ Previous Affiliation: Indian Institute of Science Education and Research Mohali,

Department of Biological Sciences, Knowledge City, Sector 81, SAS Nagar, PO Manauli, Punjab 140306, India

Ph: +91- 172-224-0266, 224-0124

Fax: +91- 172-224-0266, 224-0124

^bc^ Previous Affiliation: Indian Institute of Science Education and Research Mohali,

Department of Biological Sciences, Knowledge City, Sector 81, SAS Nagar, PO Manauli, Punjab 140306, India

Ph: +91- 172-224-0266, 224-0124

Fax: +91- 172-224-0266, 224-0124

^*^ corresponding author

**Supplementary Information**

**Table S1A. Immediate effect of cold shock on 0-6 h of egg viability**

Cold shock (0-6 h)

anova(fit_ev_cs_0-6 h)

Type III Analysis of Variance Table with Satterthwaite's method

|  | **Sum Sq** | **Mean Sq** | **NumDF** | **DenDF** | **F value** | **Pr(>F)** |
| --- | --- | --- | --- | --- | --- | --- |
| Selection (Sel) | 2 | 2 | 1 | 5.2645 | 0.3608 | 0.5730 |
| Treatment (Trt) | 33185 | 33185 | 1 | 6.0548 | 5976.7529 | **2.671e-10** |
| Sel:Trt | 2 | 2 | 1 | 4.5212 | 0.3458 | 0.5846 |

*p-*values in bold are statistically significant.

ranova(fit_ev_cs_0-6 h)

boundary (singular) fit: see? is Singular

ANOVA-like table for random-effects: Single term deletions

Model:

Egg.viability ~ Selection + Treatment + (1 | Block) + (1 | Block:Selection) + (1 | Block:Treatment) + Selection:Treatment

|  | **npar** | **logLik** | **AIC** | **LRT** | **Df** | **Pr(>Chisq)** | |
| --- | --- | --- | --- | --- | --- | --- | --- |
| <none> | 8 | -40.977 | 97.954 |  |  |  |  |
| (1 \| Block) | 7 | -40.977 | 95.954 | 0.000000 | 1 | 0.9999 |  |
| (1 \| Block:Selection) | 7 | -40.991 | 95.982 | 0.028029 | 1 | 0.8670 |  |
| (1 \| Block:Treatment) | 7 | -41.040 | 96.079 | 0.125469 | 1 | 0.7232 |  |
| Warning message: |  |  |  |  |  |  |  |

In checkConv(attr(opt, "derivs"), opt$par, ctrl = control$checkConv, :Model failed to converge with max|grad| = 0.172747 (tol = 0.002, component 1

**Table S1B. Increased egg viability at 24-30 h post cold shock in the selected populations**

>anova(fit_ev_cs_24-30 h)

Type III Analysis of Variance Table with Satterthwaite's method

# **Sum Sq Mean Sq NumDF DenDF F value Pr(>F)**

| Selection (Sel) | 1881.8 | 1881.8 | 1 | 4.0009 | 21.281 **0.009927** |
| --- | --- | --- | --- | --- | --- |
| Treatment (Trt) | 4052.7 | 4052.7 | 1 | 4.0051 | 45.831 **0.002473** |
| Sel:Trt | 2136.2 | 2136.2 | 1 | 4.0002 | 24.158 **0.007956** |

*p-*values in bold are statistically significant.

ranova(fit_ev_cs_24)

boundary (singular) fit: see? is Singular

ANOVA-like table for random-effects: Single term deletions

**Model:** Egg.viability ~ Selection + Treatment + (1 | Block) + (1 | Block:Selection) + (1 | Block:Treatment) + Selection:Treatment

|  | **npar** | **logLik** | **AIC** | **LRT** | **Df** | | **Pr(>Chisq)** |
| --- | --- | --- | --- | --- | --- | --- | --- |
| <none> | 8 | 65.531 | -147.06 |  |  | |  |
| (1 \| Block) | 7 -65.531 145.06 0.00059 | | | | | 1 | 0.9807 |
| (1 \| Block:Selection) | 7 -65.538 145.07 0.01371 | | | | | 1 | 0.9068 |
| (1 \| Block:Treatment) | 7 -65.970 145.94 0.87831 | | | | | 1 | 0.3487 |

| pairs(emmeans(fit_ev_cs_24, ~Selection*Treatment)) | | | | |  |
| --- | --- | --- | --- | --- | --- |
| **contrast** | **estimate** | **SE** | **df** | **t.ratio p.value** | |
| FCB cold shock - FSB cold shock | -41.2 | 6.13 | 7.97 | -6.728 **0.0007** | |
| FCB cold shock - FCB no shock | -64.0 | 7.66 | 6.91 | -8.357 **0.0003** | |
| FCB cold shock - FSB no shock | -63.9 | 7.80 | 7.14 | -8.192 **0.0003** | |
| FSB cold shock - FCB no shock | -22.8 | 7.80 | 7.14 | -2.918 0.0820 | |
| FSB cold shock - FSB no shock | -22.7 | 7.66 | 6.91 | -2.959 0.0798 | |
| FCB no shock - FSB no shock | 0.1 | 6.13 | 7.97 | 0.016 1.0000 | |

*p-*values in bold are statistically significant.

Degrees-of-freedom method: kenward-roger

P value adjustment: tukey method for comparing a family of 4 estimates

# **Table S1C. Higher mating frequency in the selected populations**

anova(fit_mr_cs)

Type III Analysis of Variance Table with Satterthwaite's method

|  | **Sum**  **Sq** | **Mean**  **Sq** | **Num**  **DF** | **Den**  **DF** | **F value** | **Pr(>F)** |
| --- | --- | --- | --- | --- | --- | --- |
| Selection (Sel) | 292.6 | 292.6 | 1 | 3.9998 | 22.208 | **0.009223** |
| Treatment (Trt) | 390.1 | 390.1 | 1 | 3.9999 | 29.611 | **0.005537** |
| Sel:Trt | 3537.8 | 3537.8 | 1 | 4.0001 | 268.530 | **8.117e-05** |

*p-*values in bold are statistically significant.

ranova(fit_mr_cs)

ANOVA-like table for random-effects: Single term deletions

Model:

Matings ~ Selection + Treatment + (1 | Block) + (1 | Block:Selection)

+ (1 | Block:Treatment) + Selection:Treatment

|  | **npar** | **logLik** | **AIC** | **LRT** | **Df** | **Pr(>Chisq)** |
| --- | --- | --- | --- | --- | --- | --- |
| <none> | 8 | -67.088 | 150.18 |  |  |  |
| (1 \| Block) | 7 | -68.157 | 150.31 | 2.1388 | 1 | 0.143616 |
| (1 \| Block:Selection) | 7 | -70.436 | 154.87 | 6.6979 | 1 | **0.009653** |
| (1 \| Block:Treatment) | 7 | -69.779 | 153.56 | 5.3828 | 1 | **0.020336** |

*p-*values in bold are statistically significant.

pairs(emmeans(fit_mr_cs, ~Selection*Treatment))

| **contrast** | **estimate** | **SE** | **df** | **t.ratio** | **p.value** |
| --- | --- | --- | --- | --- | --- |
| FCB cold shock - FSB cold shock | -60.2 | 7.31 | 4.41 | -8.233 | **0.0028** |
| FCB cold shock - FCB no shock | 5.6 | 6.14 | 4.60 | 0.913 | 0.8003 |
| FCB cold shock - FSB no shock | -1.4 | 9.27 | 7.74 | -0.151 | 0.9987 |
| FSB cold shock - FCB no shock | 65.8 | 9.27 | 7.74 | 7.102 | **0.0005** |
| FSB cold shock - FSB no shock | 58.8 | 6.14 | 4.60 | 9.583 | **0.0012** |
| FCB no shock - FSB no shock | -7.0 | 7.31 | 4.41 | -0.957 | 0.7788 |

*p-*values in bold are statistically significant.

Degrees-of-freedom method: kenward-roger

P value adjustment: tukey method for comparing a family of 4 estimates

**Table S2A: Immediate effect of heat shock on egg viability of 0-6 h**

>anova(fit_ev_hs_0-6 h)

Type III Analysis of Variance Table with Satterthwaite's method

|  | **Sum**  **Sq** | **Mean**  **Sq** | **Num**  **DF** | **Den**  **DF** | **F value** | **Pr(>F)** |
| --- | --- | --- | --- | --- | --- | --- |

| Selection (Sel) | 22 | 22 | 1 | 16 | 1.2643 | 0.2774 |  |
| --- | --- | --- | --- | --- | --- | --- | --- |
| Treatment (Trt) | 37732 | 37732 | 1 | 16 | 2146.12 | **<2e-16** |  |
| Sel:Trt | 3 | 3 | 1 | 16 | 0.1989 | 0.6616 |  |

*p-*values in bold are statistically significant.

ranova(fit_ev_hs_0-6 h)

boundary (singular) fit: see ?isSingular boundary (singular) fit: see ?isSingular boundary (singular) fit: see ?isSingular

ANOVA-like table for random-effects: Single term deletions

**Model:**

Egg.viability ~ Selection + Treatment + (1 | Block) + (1 | Block:Selection) + (1 | Block:Treatment) + Selection:Treatment

|  | **npar** | **logLik** | **AIC** | **LRT** | **Df** | **Pr(>Chisq)** |
| --- | --- | --- | --- | --- | --- | --- |
| <none> | 8 | -48.857 | 113.71 |  |  |  |
| (1 \| Block) | 7 | -48.857 | 111.71 | 0 | 1 | 1 |
| (1 \| Block:Selection) | 7 | -48.857 | 111.71 | 0 | 1 | 1 |
| (1 \| Block:Treatment) | 7 | -48.857 | 111.71 | 0 | 1 | 1 |
|  |  |  |  |  |  |  |

# **Table S2B. Selected population have heightened egg viability at 24-30 h post heat shock**

>anova(fit_ev_hs_24-30h )

Type III Analysis of Variance Table with Satterthwaite's method

|  | **Sum Sq** | **Mean Sq** | **NumDF** | **DenDF** | **F value** | **Pr(>F)** |
| --- | --- | --- | --- | --- | --- | --- |
| Selection (Sel) | 63.00 | 63.00 | 1 | 8.0000 | 30.290 | **0.0005713** |
| Treatment (Trt) | 1186.19 | 1186.19 | 1 | 4.0016 | 570.328 | **1.817e-05** |
| Sel:Trt | 66.62 | 66.62 | 1 | 8.0000 | 32.029 | **0.0004762** |

*p-*values in bold are statistically significant.

ranova(fit_ev_hs_24-30 h)

boundary (singular) fit: see ?isSingular

ANOVA-like table for random-effects: Single term deletions

Model:

Egg.viability ~ Selection + Treatment + (1 | Block) + (1 | Block:Selection) + (1 | Block:Treatment) + Selection:Treatment

| <none> | **npar**  8 | | **logLik**  -43.035 | | **AIC**  102.07 | | **LRT** | | **Df** | **Pr(>Chisq)** | |
| --- | --- | --- | --- | --- | --- | --- | --- | --- | --- | --- | --- |
| (1 \| Block) | 7 | | -43.037 | | 100.07 | | 0.0044 | | 1 | 0.947229 | |
| (1 \| Block:Selection) | 7 | | -43.035 | | 100.07 | | 0.0000 | | 1 | 0.999801 | |
| (1 \| Block:Treatment) | 7 | | -48.267 | | 110.53 | | 10.464 | | 1 | **0.001217** | |
| *p-*values in bold are statistically significant. pairs(emmeans(fit_ev_hs_24-30 h, ~Selection*Treatment)) | | | | | | | | | | | |
| **contrast** | | **estimate** | | **SE** | | **df** | | **t.ratio** | | | **p.value** |
| FCB heat shock - FSB heat shock | | -7.2 | | 0.912 | | 8.00 | | -7.893 | | | **0.0002** |
| FCB heat shock - FCB no shock | | -65.5 | | 2.669 | | 4.49 | | -24.541 | | | **<.0001** |
| FCB heat shock - FSB no shock | | -65.4 | | 2.669 | | 4.49 | | -24.504 | | | **<.0001** |
| FSB heat shock - FCB no shock | | -58.3 | | 2.669 | | 4.49 | | -21.844 | | | **<.0001** |
| FSB heat shock - FSB no shock | | -58.2 | | 2.669 | | 4.49 | | -21.806 | | | **<.0001** |
| FCB no shock - FSB no shock | | 0.1 | | 0.912 | | 8.00 | | 0.110 | | | 0.9995 |

*p-*values in bold are statistically significant.

Degrees-of-freedom method: kenward-roger

P value adjustment: tukey method for comparing a family of 4 estimates

# **Table S2C. Increased mating frequency in the selected populations**

anova(fit_mr_hs)

Type III Analysis of Variance Table with Satterthwaite's method

|  | **Sum**  **Sq** | **Mean**  **Sq** | **Num**  **DF** | **Den**  **DF** | **F value** | **Pr(>F)** |
| --- | --- | --- | --- | --- | --- | --- |
| Selection (Sel) | 952.2 | 952.2 | 1 | 8 | 8.1246 | **0.02147** * |
| Treatment (Trt) | 1.1 | 1.1 | 1 | 8 | 0.0094 | 0.92514 |
| Sel:Trt | 231.2 | 231.2 | 1 | 8 | 1.9727 | 0.19778 |

*p-*values in bold are statistically significant.

ranova(fit_mr_hs)

boundary (singular) fit: see? is Singular

ANOVA-like table for random-effects: Single term deletions

Model:

Matings ~ Selection + Treatment + (1 | Block) + (1 | Block:Selection)

+ (1 | Block:Treatment) + Selection:Treatment

|  | **npar** | **logLik** | **AIC** | **LRT** | **Df** | **Pr(>Chisq)** |
| --- | --- | --- | --- | --- | --- | --- |
| <none> | 8 | -68.297 | 152.59 |  |  |  |
| (1 \| Block) | 7 | -68.297 | 150.59 | 0.000 | 1 | 1.0000 |
| (1 \| Block:Selection) | 7 | -68.297 | 150.59 | 0.000 | 1 | 1.0000 |
| (1 \| Block:Treatment) | 7 | -69.384 | 152.77 | 2.173 | 1 | 0.1405 |

**Table S3.** Summary of the results from a two-factor mixed model ANOVA on adult survival in male (**S3A**) and female (**S3B**) post cold shock. And results from a two-factor mixed model ANOVA and on adult survival in male (**S3C**) and female (**S3D**) post heat shock employing selection regime (FSB and FCB) as the fixed factor crossed with blocks (1-5) as a random factor. *p-*values in bold are statistically significant.

| **Trait** | **Effect** | **SS** | **MS**  **Num** | **DF Num** | **DF Den** | ***F* ratio** | ***P*** |
| --- | --- | --- | --- | --- | --- | --- | --- |
| (**S3A**) | Selection (Sel) | 0.566 | 0.566 | 1 | 4 | 40.209 | 0.003 |
| Males | Block (Blk) | 0.296 | 0.074 | 4 | 4 | 5.245 | 0.069 |
| cold shock | Sel×Blk | 0.056 | 0.014 | 4 | 20 | 2.138 | 0.114 |
| (**S3B**) | Selection (Sel) | 0.637 | 0.637 | 1 | 4 | 52.076 | 0.002 |
| Females | Block (Blk) | 0.268 | 0.067 | 4 | 4 | 5.486 | 0.064 |
| cold shock | Sel×Blk | 0.049 | 0.012 | 4 | 20 | 2.240 | 0.101 |
| (**S3C**) | Selection (Sel) | 0.154 | 0.154 | 1 | 4 | 69.272 | 0.001 |
| Males | Block (Blk) | 0.227 | 0.057 | 4 | 4 | 25.460 | 0.004 |
| heat shock | Sel×Blk | 0.009 | 0.002 | 4 | 20 | 0.206 | 0.932 |
| (**S3D**) | Selection (Sel) | 0.105 | 0.105 | 1 | 4 | 60.146 | 0.001 |
| females | Block (Blk) | 0.026 | 0.006 | 4 | 4 | 3.737 | 0.115 |
| heat shock | Sel×Blk | 0.007 | 0.002 | 4 | 20 | 0.630 | 0.647 |

# **Table S4.** Estimates and 95% confidence intervals (CIs) for relative hazard rates (i. e. the exponent of the coefficients) corresponding to various fixed parameters of the Cox’s proportional hazards model for **survivorship post infection**. Hazard rates are expressed relative to the default level of that fixed factor. The default level for selection regime is FCB, while the default level for sex is female. Hazard rates significantly greater than 1 correspond to poorer survivorship relative to the default level.

| **Survivorship post infection** | | | |
| --- | --- | --- | --- |
| **Fixed factors** | | | |
| **Coefficient** | **Hazard ratio** | **Lower CL** | **Upper CL** |
| SelectionFSB | 0.8149 | 0.6604 | 1.0056 |
| SexMale | 0.9528 | 0.6963 | 1.3037 |
| SelectionFSB:SexMale | 1.0309 | 0.7578 | 1.4025 |
|  | | | |
| **Random factors** | | | |
| **Group** | **Variance** |  |  |
| Block/Sex/Selection | 0.0013 |  |  |
| Block/Sex | 0.0343 |  |  |
| Block | <0.0001 |  |  |

**Table S5.** Estimates and 95% confidence intervals (CIs) for relative hazard rates (i. e. the exponent of the coefficients) corresponding to various fixed parameters of the Cox’s proportional hazards model for desiccation resistance of males (A) and females (B). Hazard rates are expressed relative to the default level of that fixed factor. The default level for selection regime is FCB. Hazard rates significantly greater than 1 correspond to poorer survivorship relative to the default level.

| **(S5A) Male desiccation resistance** | | | |
| --- | --- | --- | --- |
| **Fixed factors** | | | |
| **Coefficient** | **Hazard ratio** | **Lower CL** | **Upper CL** |
| SelectionFSB | 1.1600 | 0.3261 | 4.1262 |
|  | | | |
| **Random factors** | | | |
| **Group** | **Variance** |  |  |
| Block/Selection | 1.0324 |  |  |
| Block | 0.0714 |  |  |
|  | | | |
| **(S5B) Female desiccation resistance** | | | |
| **Fixed factors** | | | |
| **Coefficient** | **Hazard ratio** | **Lower CL** | **Upper CL** |
| SelectionFSB | 0.5393 | 0.2760 | 1.0539 |
|  | | | |
| **Random factors** | | | |
| **Group** | **Variance** |  |  |
| Block/Selection | 0.2770 |  |  |
| Block | 0.2889 |  |  |

**Table S6.** Estimates and 95% confidence intervals (CIs) for relative hazard rates (i. e. the exponent of the coefficients) corresponding to various fixed parameters of the Cox’s proportional hazards model for starvation resistance of males (A) and females (B). Hazard rates are expressed relative to the default level of that fixed factor. The default level for selection regime is FCB. Hazard rates significantly greater than 1 correspond to poorer survivorship relative to the default level.

| **(S6A) Male starvation resistance** | | | |
| --- | --- | --- | --- |
| **Fixed factors** | | | |
| **Coefficient** | **Hazard ratio** | **Lower CL** | **Upper CL** |
| SelectionFSB | 2.0405 | **1.1831** | **3.5192** |
|  | | | |
| **Random factors** | | | |
| **Group** | **Variance** |  |  |
| Block/Selection | 0.1800 |  |  |
| Block | 0.6627 |  |  |
|  | | | |
| **(S6B) Female starvation resistance** | | | |
| **Fixed factors** | | | |
| **Coefficient** | **Hazard ratio** | **Lower CL** | **Upper CL** |
| SelectionFSB | 1.5543 | **1.0619** | **2.2749** |
|  | | | |
| **Random factors** | | | |
| **Group** | **Variance** |  |  |
| Block/Selection | 0.0793 |  |  |
| Block | 0.0004 |  |  |
